# Immunosuppression and Multiple Primary Malignancies in Kidney-Transplanted Patients: A Single-Institute Study

**DOI:** 10.1155/2015/183523

**Published:** 2015-06-22

**Authors:** Michele L. Santangelo, Carmen Criscitiello, Andrea Renda, Stefano Federico, Giuseppe Curigliano, Concetta Dodaro, Alessandro Scotti, Vincenzo Tammaro, Armando Calogero, Eleonora Riccio, Antonio Pisani, Nicola Carlomagno

**Affiliations:** ^1^Department of Advanced BioMedical Sciences, Operative Unit of General Surgery & Transplants, University of Naples Federico II, 80131 Naples, Italy; ^2^Division of Early Drug Development for Innovative Therapies, European Institute of Oncology, 20141 Milan, Italy; ^3^Department of Public Medicine, Operative Unit of Nephrology, University of Naples Federico II, 80131 Naples, Italy

## Abstract

Immunodeficiency is associated with higher cancer incidence. However, it is unknown whether there is a link between immunodeficiency and development of multiple primary malignancies. In the present study we analyse this link focusing on kidney-transplanted patients, as they are at higher risk of developing cancer due to the chronic assumption of immunosuppressants. We followed up 1200 patients who underwent kidney transplantation between 1980 and 2012. A total of 77/1200 kidney-transplanted patients developed cancer and 24 of them developed multiple cancers. Most multiple cancers were synchronous with a nonsignificant association between cancer and rejection episodes. In the general cancer population, one-ninth of patients are at higher risk of developing a second tumor over a lifetime; hence it would be reasonable to conclude that, from a merely theoretical and statistical viewpoint, long-term transplanted patients potentially have a higher risk of developing MPMs. However, data did not confirm this assumption, probably because these patients die before a second primary malignancy appears. Despite many observations on the increased incidence of different tumor types in immunodeficient patients and despite immunosuppression certainly being a predisposing factor for the multicancer syndrome, data so far are not robust enough to justify a correlation between immunodeficiency and multiple primary malignancies in transplanted patients.

## 1. Introduction

Renal transplantation is the gold standard procedure for patients with end-stage renal diseases. The increasing success of such an approach is partly due to the use of increasingly active immunosuppressive drugs, which have largely lowered the rate of rejection and improved outcome [1]. However, the chronic use of immunosuppressive drugs leads to an increased cancer incidence [2]. A link between cancer development and immunosuppression in transplanted patients is well recognized. Over the past decades, a growing body of evidence has emerged demonstrating the dual role of the immune system in cancer, being involved both in tumor development (via chronic inflammation through the innate immune system) and in tumor elimination and control (through the adaptive immune system) [3]. As an example, renal cell carcinoma has been traditionally considered immunogenic, as it does occur at a higher incidence in immunosuppressed patients [4]. Also, this tumor type is traditionally considered more responsive to immunotherapy. Therefore, it is now well recognized that immunosuppressive drugs used in transplanted patients may induce immune defects, thus compromising the immune response and facilitating the development of a secondary immunodeficiency (ID) which can ultimately ease cancer onset [5]. Also, the incidence of multiple primary malignancies (MPMs) is increasing in the general population and it is expected to further increase in the coming years. The definition of MPMs requires that each tumor be a solid tumor, have a histopathological diagnosis of malignancy, be topographically distinct from another one, and not include tumor that are metastases of the primary. In terms of time, they are classified as simultaneous (i.e., both tumors appear at the same time), synchronous (i.e., the second tumor appears within six months from the first tumor), and metachronous (i.e., the second tumor appears more than six months after the first tumor) [6]. Having said that, it has to be considered that should a transplanted patient develop a tumor, recover from it, and continue to receive immunosuppressive treatment, the risk of developing MPMs is potentially higher as compared to the general population [7]. It is in this perspective that the problem of MPMs in transplanted patients should be considered. In this paper we focus on the link between secondary immunodeficiency and the onset of MPMs in transplanted patients, who are—by definition—patients at higher risk of developing cancer due to the chronic assumption of immunosuppressants.

To test the relationship between immunosuppression and MPMs, we specifically selected kidney-transplanted patients for several reasons. Firstly, among solid organ transplanted patients, kidney recipients represent the most numerous group, with the longest follow-up (kidney transplantation was the first solid organ transplant carried out; from a single cadaver donor it is generally possible to obtain two kidneys for two different kidney recipients; living kidney donation is a perfectly codified procedure which is carried out worldwide). Therefore, this is a representative population. Secondly, the median overall survival of kidney transplant recipients is long (more than 10 years) and, accordingly, so is the exposure to immunosuppressive drugs; hence, in these patients it is possible to evaluate whether there is a correlation between immunosuppression and cancer(s) development over an adequate timeframe. Thirdly, after kidney transplantation, immunosuppressants are generally used at full dosage, thus making it possible to evaluate their real effects on tumorigenesis. Last but not least, in this population, transplanted organ failure does not inevitably lead to death, as it is possible to return to dialysis. Therefore, in these patients, the natural history of the disease may be also evaluated after reduction/modification/interruption of immunosuppressive drugs. For all the above-mentioned reasons, we analyzed a consecutive series of patients undergoing kidney transplantation at our institute.

## 2. Material and Methods

Through the analysis of medical records collected in our department, we retrospectively examined 1200 kidney-transplanted patients (745 male and 455 female) followed up at Federico II University between 1980 and 2012. The median follow-up was 10 years and the average graft survival 8 years. Patients' age ranged between 18 and 65 years and they were homogenous for donor/recipient immunology (e.g., mismatch index), number of rejection events, and immunosuppressive therapy. In our population, different drugs and associations were used taking advantage of their different mechanisms of actions: corticosteroids (the oldest immunosuppressants), azathioprine (an old antiproliferative immunosuppressive drug), calcineurin inhibitors (cyclosporine and tacrolimus are the most used as maintenance therapy; they primarily suppress the activation of T lymphocytes by inhibiting the production of cytokines, specifically IL-2), basiliximab (an IL-2 receptor antagonist generally used as induction therapy), mycophenolate (a new antiproliferative agent that interferes with DNA replication, producing cytostatic effects on both T and B cells; it is generally used as a “third agent” in triple immunosuppressive regimens), mammalian target of rapamycin inhibitors (also called m-TOR inhibitors; they usually represent an alternative to the long-term calcineurin inhibitor-based regimen and its side effects). Drugs and associations have varied over time. In general we used corticosteroids and azathioprine from 1980 to 1984; corticosteroids, calcineurin inhibitors, and azathioprine from 1984 to 1998; corticosteroids, calcineurin inhibitors, basiliximab, mycophenolate, and mammalian target of rapamycin from 1998 to 2013. We did not at any time use thymoglobulin.

## 3. Results

**Figure 1:**
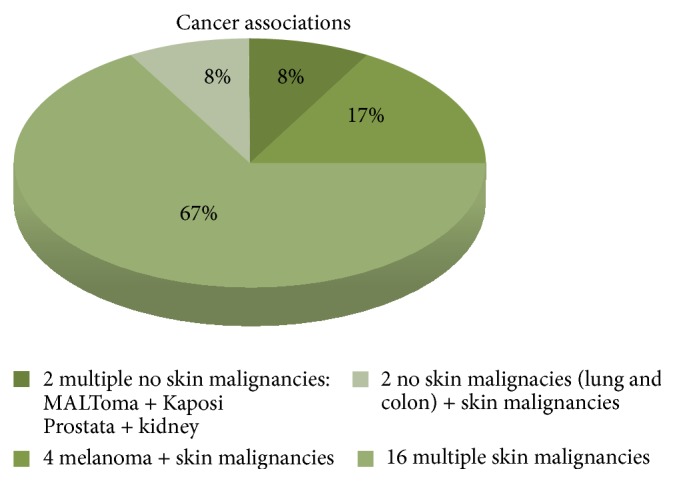
Cancers associations among 24 patients in 1200 kidney-transplant patients

**Figure 2:**
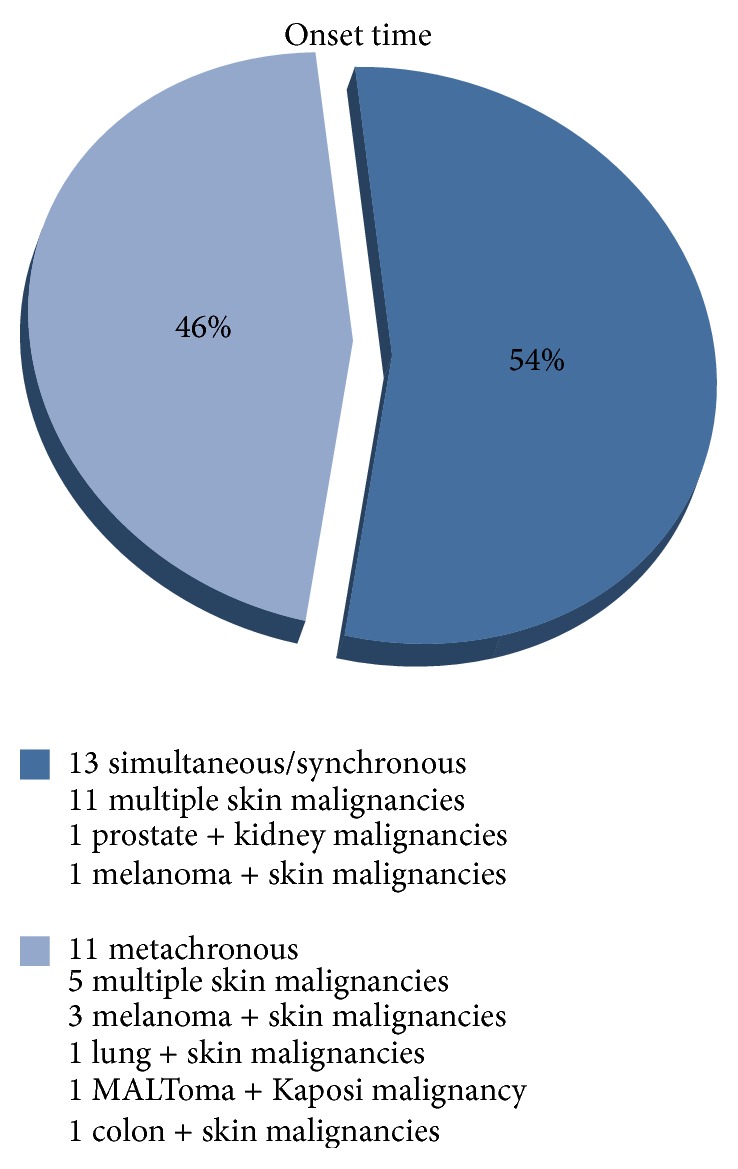
Onset time of MPMs among 24 patients in 1200 kidney-transplant patients

Among 1200 kidney-transplanted patients, 77 patients (6.4%) [57 males (4.7%) and 20 females (1.7%)], developed a cancer. A total of 53 patients (4.4% of global series and 69% of cancer patients) developed a single cancer. A total of 20 patients were diagnosed with a skin cancer (including melanoma) and 33 patients with no skin cancer, representing 1.7% and 2.7% of global series and 26% and 43% of cancer patients, respectively. It is worth noting that 24 patients (2% of global series and 31% of cancer patients) presented with multiple cancers: 20 multiple skin cancers (including melanoma) and 4 solid MPMs, representing 1.6% and 0.3% of global series and 25.9% and 5.1% of cancer patients, respectively (Table 1). With regard to the latter group, we observed the following associations: prostate/kidney cancer (synchronous, surgically treated, 2-year disease-free interval); Kaposi sarcoma/gastric MALToma (metachronous, medical and surgical treatment, 1-year disease-free interval, exitus at 18 months); lung cancer/squamous skin carcinoma (metachronous, medical and surgical treatment, disease-free at 8 months, exitus at 14 months), and colon cancer/squamous skin carcinoma (metachronous, surgically treated, 18-month disease-free interval). Multiple cancer associations and their onset time are detailed in Figures 1 and 2. Data on the association between cancer and rejection episodes are not significant: rejection episodes were only found in four patients and only one of them developed a second cancer.

## 4. Discussion

**Figure 3:**
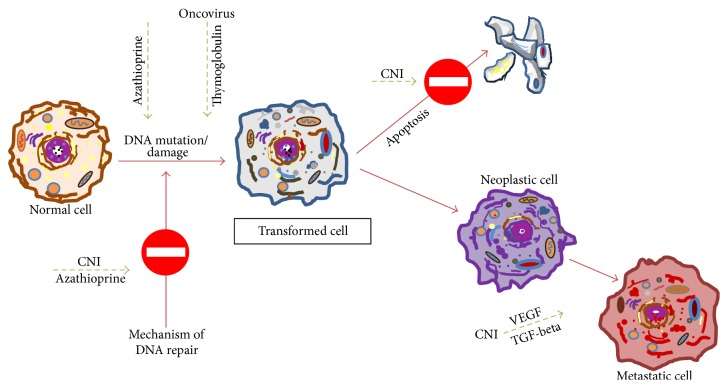
Schematic representation of some oncogenic mechanisms of calcineurin inhibitors (CNI), azathioprine, and thymoglobulin.

Today the high standards in surgical, anaesthesiological, and intensive-care procedures as well as in the clinical management of patients undergoing transplantation enable extremely positive results to be obtained in terms of short- and medium-term survival for both organs and patients [8–13]. However, these results have been partially nullified by the long-term complications reported in these patients, especially the development of cancer. Incidence, aggressiveness, and worse prognosis of tumors appear to be remarkably increased in this group of patients as compared to the general population of corresponding age [7, 14–16]. It has been calculated that tumor prevalence at 10 years after kidney transplant ranges from 20% to 30%, with peaks also over 45% at 20 years [16–21]. Among organ transplant recipients the main factor facilitating cancer onset is certainly the immunosuppressive treatment. Indeed, the neoplastic risk is also increased in people treated with immunosuppressants for reasons other than transplantation [20]. Besides the indirect oncogenic effect exerted by all immunosuppressive drugs, which alter the immune response, recent studies have shown that some immunosuppressants (i.e., calcineurin inhibitors, azathioprine, and thymoglobulin) exert a direct oncogenic effect. Calcineurin inhibitors promote oncogenesis, neoplastic growth, and metastasization by inhibiting DNA repair and apoptosis and by stimulating the production of transforming growth factor-beta (TGF-beta) and vascular endothelial growth factor (VEGF). Azathioprine and its derivatives are able to increase DNA damage caused by UV and to inhibit DNA repair. Thymoglobulin seems to foster genetic mutations induced by oncoviruses (Figure 3). In this context, it is worth noting that, in transplanted patients, some tumor types may show a regression if immunosuppressive therapy is withdrawn or changed/enriched with drugs such as mTOR inhibitors and mycophenolate [22, 23]. However, while the use of mycophenolate was associated with a reduced cancer incidence, probably because its administration is correlated to calcineurin inhibitor dose reduction, mTOR inhibitors have shown a direct antineoplastic effect. These properties—together with a reduced nephrotoxicity—have led to an extended use of these drugs [24–35].

**Figure 4:**
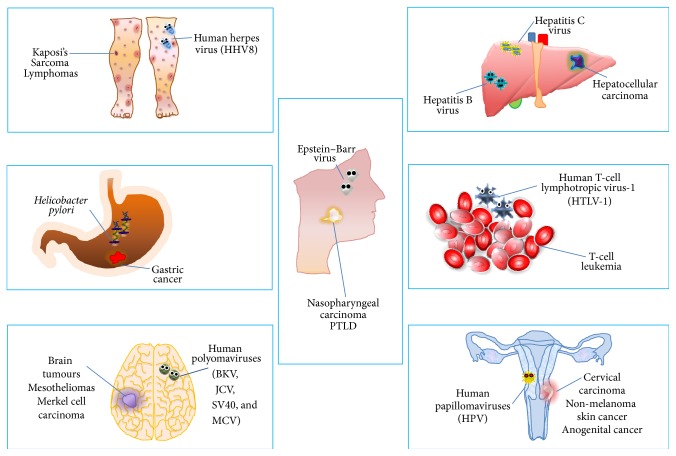
Oncoviruses and potentially related cancers.

However, in transplanted patients, immunosuppressive therapy is essential to avoid graft rejection, which ultimately results in reduced morbidity and mortality. There is a huge variability among different classes of immunosuppressive drugs, which work through different mechanisms on the immune system. When analyzing the association between immunosuppressive therapy and increased cancer risk, different aspects have to be considered, such as the duration of immune suppressive therapy, the intensity of treatment, and the drug(s) used. Yet, such a huge variability in terms of clinical studies' fragmentation, uniqueness of each single patient, different therapeutic approaches in different transplant centers, the switch from one immunosuppressive protocol to another, and—last but not least—the pressure exercised by pharmaceutical firms has led to contrasting results. Also, it is worth remembering that the lifespan of transplanted patients is longer; accordingly the time frame these people stay on immunosuppressive treatment is longer too, with augmented exposure to oncogenic factors and viral infections (Figure 4). Furthermore, transplanted patients nowadays have a longer life expectancy and may reach the age at which the neoplastic risk is naturally higher, when the transplant is not already performed in aged people [19]. Overall, the increased cancer risk after renal transplantation is now well recognized [36–40]. So, the association between pharmacological immune suppression and increased risk of cancer continues to be a much-discussed topic [41]. It has been calculated that if malignant tumors carried a lower mortality rate and were more uniformly distributed in the general population, we could still expect to find that 1 in 9 cancer patients would develop a second cancer over a lifetime and that within this group 1 in 27 patients will probably develop a third primary cancer [6, 41–46]. This statistical projection obviously refers to the general population. Therefore, it would be logical to conclude that, from a merely theoretical and probabilistic point of view, immunocompromised patients potentially have a higher risk of developing MPMs [6, 24, 42, 46–48]. Transplanted patients treated with immunosuppressants may develop multiple cancers in three different conditions: (1) patients with a previous diagnosis of cancer who undergo transplantation and, afterwards, present with a new cancer during follow-up; (2) patients with a previous diagnosis of cancer who undergo transplantation and then present with a new cancer transmitted by the donor; (3) patients developing MPMs after transplantation (those reported in our study). But real life differs from theory, even when the theory has valid bases. To the best of our knowledge, only one study has specifically looked at the incidence of MPMs in transplanted patients [49]. In this study, transplanted patients did not show a statistically significant higher risk of developing MPMs as compared to the corresponding general population. Also, our experience together with a careful review of the literature does not support the hypothesis that immunocompromised patients are more likely to develop MPMs. The reasons for this might simply lie in the fact that kidney-transplanted patients probably die before a new “second primary malignancy” appears or, alternatively, returning to dialysis, they interrupt immunosuppressive therapy, thus limiting the exposure to oncogenic effects of such drugs over time. Furthermore, it is very hard to find transplanted patients surviving a first cancer who keep on taking immune suppressive treatment long enough to develop a second primary cancer as a consequence of iatrogenic immune deficiency. Indeed, until recently, kidney transplant recipients who developed a tumor were treated according to medical/surgical approaches, which included—among others—immunosuppressant withdrawal and, accordingly, return to dialysis. Nowadays, the chance to have recourse to immunosuppressants such as mycophenolate and mTOR inhibitors has allowed a large number of kidney-transplanted patients who develop a tumor to recover by maintaining the function of the transplanted organ. Hence, we are observing a group of kidney-transplanted patients at higher risk of developing a second tumor, as they recovered from the first one without interrupting immunosuppressive therapy. However, the follow-up of these patients is still limited; therefore, it is not yet possible to evaluate the actual incidence of second tumors.

## 5. Conclusions

Despite many observations regarding the increased incidence of different tumor types in immunosuppressed patients and despite the fact that immunosuppression is a predisposing factor for multicancer syndrome, at least theoretically, so far there are no significant statistical data indicating a clear correlation between immunosuppression and MPMs. We may therefore assume that it is hard to diagnose a second cancer in immunocompromised patients because of their shorter life expectancy. From the few reports found in the literature and from our experience, we can conclude that MPMs in immunosuppressed patients are more frequently simultaneous/synchronous, usually have a viral etiology, and regard the same organ or tissue, the skin is the most affected tissue with a predominance of spinocellular carcinomas over basocellular carcinomas (exactly the opposite of what is observed in the general population), and at least one cancer is readily detectable (e.g., skin cancer), thereby facilitating an early diagnosis and treatment. It is our opinion that the treatment of MPMs in immunosuppressed patients should be as intensive as possible, in order to obtain a complete recovery. Moreover, it might be useful to suspend the immunosuppressive treatment or switch to other drugs such as m-TOR inhibitors; this therapeutic approach has so far yielded good results. In conclusion, available clinical and epidemiological data allow immunosuppression to be considered as a cancer risk factor. However, so far there is no sufficient evidence to conclude that immunosuppression eases the onset of MPMs. Hence, even if MPMs do not seem to be a real problem today, they may become an important issue in the near future, when new treatments and stricter follow-up guarantee longer life expectancy in immunosuppressed patients diagnosed with cancer. Therefore, in potentially immunocompromised patients (e.g., kidney transplant candidates), great relevance must be given to preventive measures against oncoviral infections (e.g., a vaccination program, as has already been established for HBV and HPV); implementation of procedures aiming at reducing the exposure to environmental oncogenic factors (e.g., drugs, cigarette smoking, alcohol, sun exposure, etc., as is already recommended to our patients); strict follow-up programs with special attention to apparatus/organs (e.g., genitourinary, skin, thyroid, liver, blood, and bones) at higher cancer risk in such patients and—last but not least—it is important to try to reduce the dosage of immunosuppressive drugs as much as possible (especially for calcineurin inhibitors, azathioprine, and thymoglobulin, for which a direct oncogenic effect has been proven), without exposing the patient to the risk of graft rejection. This paradigm should aim at immunomodulation rather than immunosuppression, which might be the true gold standard of such a therapy.

## Figures and Tables

**Table 1:** Characteristics of patients with MPMs.

Patient	Sex	Year of kidney transplant	Patient's age at transplant	Immunosuppressive drugs used	Acute rejection events	Type of first tumor	Date of first tumor	Type of second tumor	Date of second tumor	Return to dialysis (year)	Status (last follow-up)
1	M	1995	39	CCS + CyA + Myc	No	BCC	2001	Melanoma	2006	No	Alive (2012)
2	M	1987	25	CCS + CyA	No	SCC	2008	SCC	2008	Yes (2010)	Alive (2012)
3	M	2001	64	CCS + CyA + Myc	No	BCC	2006	SCC	2006	Yes (2011)	Alive (2012)
4	M	2003	62	CCS + FK > > Rap + CCS	No	Prostate Ca	2010	Kidney Ca	2010	No	Alive (2012)
5	M	2001	55	CCS + CyA + Myc	No	BCC	2002	SCC	2002	No	Alive (2010)
6	M	1988	45	CyA > Rap + CCS	No	Kaposi	2004	Gastric MALToma	2005	Yes (2006)	Dead (2006)
7	M	2001	55	CCS + FK + Myc	No	BCC	2003	SCC	2003	No	Alive (2012)
8	M	1992	42	CCS + CyA	No	SCC	2005	BCC + SCC	2005	No	Alive (2012)
9	M	1997	39	CyA + Aza	No	SCC	2003	Melanoma	2012	No	Alive (2012)
10	M	1995	51	CCS + CyA + Myc	No	BCC	2005	BCC + SCC	2010/2011	No	Alive (2012)
11	F	2004	53	CCS + CyA > > Rap + CCS	No	Lung Ca	2005	SCC	2006	Yes (2007)	Dead (2007)
12	M	1998	56	CCS + CyA	No	SCC	1999	BCC	2007	No	Alive (2012)
13	M	1992	18	CCS + CyA + Myc	Yes	BCC	2000	SCC	2000	Yes (2008)	Alive (2012)
14	M	2001	61	CCS + Rap + Myc	No	SCC	2006	SCC	2007	No	Alive (2007)
15	M	1989	29	CyA + Aza > > Rap + CCS	No	BCC	2007	SCC	2007	No	Alive (2012)
16	M	2005	43	CCS + CyA	No	Melanoma	2008	BCC	2008	No	Alive (2012)
17	M	1999	59	CCS + CyA + Myc	No	BCC	2004	SCC	2004	No	Alive (2011)
18	M	1986	46	CCS + CyA	No	SCC	1997	Melanoma	2004	Yes (2004)	Dead (2007)
19	M	2000	35	CCS + CyA + Myc	No	SCC	2006	BCC	2012	No	Alive (2012)
20	M	1994	38	CCS + CYA	No	SCC	2000	SCC	2000	Yes (2010)	Alive (2012)
21	F	1999	56	CCS + CYA	No	SCC	2004	BCC	2004	No	Alive (2012)
22	F	1996	49	FK + Aza > > FK	No	BCC	2001	BCC	2001	No	Alive (2012)
23	F	1987	56	CCS + FK	No	BCC	1998	SCC	2006	Yes (2011)	Alive (2012)
24	M	2005	60	CCS + FK > > Rap > FK	No	SCC	2006	Colon Ca	2011	Yes (2012)	Alive (2012)

M: male; F: female.

CCS: corticosteroids; Aza: azathioprine; CyA: cyclosporine; FK: tacrolimus; Myc: mycophenolate and derivatives; Rap: rapamycin and derivatives.

>: switch to other drug(s).

Ca: carcinoma; SCC: squamous cell carcinoma; BCC: basal cell carcinoma; Kaposi: Kaposi sarcoma; MALToma: neoplasm of mucosa associated lymphoid tissue.

## References

[1] Rama I., Grinyó J. M. (2010). Malignancy after renal transplantation: the role of immunosuppression. *Nature Reviews Nephrology*.

[2] Engels E. A., Pfeiffer R. M., Fraumeni J. F. (2011). Spectrum of cancer risk among US solid organ transplant recipients. *The Journal of the American Medical Association*.

[3] Zitvogel L., Tesniere A., Kroemer G. (2006). Cancer despite immunosurveillance: immunoselection and immunosubversion. *Nature Reviews Immunology*.

[4] Penn I. (1988). Tumors of the immunocompromised patient. *Annual Review of Medicine*.

[5] Berg D., Otley C. C. (2002). Skin cancer in organ transplant recipients: epidemiology, pathogenesis, and management. *Journal of the American Academy of Dermatology*.

[6] Carlomagno N., Santangelo M. L., Mastromarino R., Calogero A., Dodaro C., Renda A. (2014). Rare multiple primary malignancies among surgical patients—a single surgical unit experience. *Ecancermedicalscience*.

[7] Tessari G., Naldi L., Boschiero L. (2013). Incidence of primary and second cancers in renal transplant recipients: a multicenter cohort study. *American Journal of Transplantation*.

[8] Chinen J., Anmuth D., Franklin A. R. K., Shearer W. T. (2007). Long-term follow-up of patients with primary immunodeficiencies. *The Journal of Allergy and Clinical Immunology*.

[9] de Rosa P., Santangelo M., Ferrara A. (2004). Suboptimal kidney: the experience of a single transplant unit. *Transplantation Proceedings*.

[10] Santangelo M., de Rosa P., Spiezia S. (2006). Healing of surgical incision in kidney transplantation: a single transplant center's experience. *Transplantation Proceedings*.

[11] De Rosa P., Santangelo M., Scala A. (2006). Difficult vascular conditions in kidney transplantation. *Transplantation Proceedings*.

[12] Santangelo M., Zuccaro M., de Rosa P. (2007). Older kidneys donor transplantation: five years' experience without biopsy and using clinical laboratory and macroscopic anatomy evaluation. *Transplantation Proceedings*.

[13] Santangelo M., Clemente M., Spiezia S. (2009). Wound complications after kidney transplantation in nondiabetic patients. *Transplantation Proceedings*.

[14] Dantal J., Hourmant M., Cantarovich D. (1998). Effect of long-term immunosuppression in kidney-graft recipients on cancer incidence: randomised comparison of two cyclosporin regimens. *The Lancet*.

[15] Harden P. N. (2001). Annual incidence and predicted risk of nonmelanoma skin cancer in renal transplant recipients. *Transplantation Proceedings*.

[16] Carroll R. P., Ramsay H. M., Fryer A. A., Hawley C. M., Nicol D. L., Harden P. N. (2003). Incidence and prediction of nonmelanoma skin cancer post-renal transplantation: a prospective study in Queensland, Australia. *American Journal of Kidney Diseases*.

[17] *Proceedings of the IARC Working Group on the Evaluation of Carcinogenic Risks to Humans. Epstein-Barr Virus and Kaposi's Sarcoma Herpesvirus/Human Herpesvirus 8. Lyon, France, June 1997*.

[18] EBPG Expert Group on Renal Transplantation (2002). European best practice guidelines for renal transplantation. Section IV: Long-term management of the transplant recipient. IV.1. Organization of follow-up of transplant patients after the first year. *Nephrology Dialysis Transplantation*.

[19] Sheil A. G. R. (2002). Organ transplantation and malignancy: inevitable linkage. *Transplantation Proceedings*.

[20] Gutierrez-Dalmau A., Campistol J. M. (2007). Immunosuppressive therapy and malignancy in organ transplant recipients: a systematic review. *Drugs*.

[21] Bouwes Bavinck J. N., Hardie D. R., Green A. (1996). The risk of skin cancer in renal transplant recipients in Queensland, Australia: a follow-up study. *Transplantation*.

[22] Gaumann A., Schlitt H. J., Geissler E. K. (2008). Immunosuppression and tumor development in organ transplant recipients: the emerging dualistic role of rapamycin. *Transplant International*.

[23] Robson R., Cecka J. M., Opelz G., Budde M., Sacks S. (2005). Prospective registry-based observational cohort study of the long-term risk of malignancies in renal transplant patients treated with mycophenolate mofetil. *American Journal of Transplantation*.

[24] Dantal J., Pohanka E. (2007). Malignancies in renal transplantation: an unmet medical need. *Nephrology Dialysis Transplantation*.

[25] Campistol J. M., Cuervas-Mons V., Manito N. (2012). New concepts and best practices for management of pre- and post-transplantation cancer. *Transplantation Reviews*.

[26] Rodríguez-Perálvarez M., de La Mata M., Burroughs A. K. (2014). Liver transplantation: immunosuppression and oncology. *Current Opinion in Organ Transplantation*.

[27] Asch W. S., Bia M. J. (2014). Oncologic issues and kidney transplantation: a review of frequency, mortality, and screening. *Advances in Chronic Kidney Disease*.

[28] Chapman J. R., Webster A. C., Wong G. (2013). Cancer in the transplant recipient. *Cold Spring Harbor Perspectives in Medicine*.

[29] Bottomley M. J., Harden P. N. (2013). Update on the long-term complications of renal transplantation. *British Medical Bulletin*.

[30] Piselli P., Busnach G., Fratino L. (2013). De novo malignancies after organ transplantation: focus on viral infections. *Current Molecular Medicine*.

[31] Dugue P. A., Rebolj M., Garred P., Lynge E. (2013). Immunosuppression and risk of cervical cancer. *Expert Review of Anticancer Therapy*.

[32] Hosseini-Moghaddam S. M., Soleimanirahbar A., Mazzulli T., Rotstein C., Husain S. (2012). Post renal transplantation Kaposi's sarcoma: a review of its epidemiology, pathogenesis, diagnosis, clinical aspects, and therapy. *Transplant Infectious Disease*.

[33] Ponticelli C. (2011). Present and future of immunosuppressive therapy in kidney transplantation. *Transplantation Proceedings*.

[34] Suthanthiran M., Hojo M., Maluccio M., Boffa D. J., Luan F. L. (2009). Post-transplantation malignancy: a cell autonomous mechanism with implications for therapy.. *Transactions of the American Clinical and Climatological Association*.

[35] Karran P., Attard N. (2008). Thiopurines in current medical practice: molecular mechanisms and contributions to therapy-related cancer. *Nature Reviews Cancer*.

[36] Piselli P., Serraino D., Segoloni G. P. (2013). Risk of de novo cancers after transplantation: results from a cohort of 7217 kidney transplant recipients, Italy 1997–2009. *European Journal of Cancer*.

[37] Pedotti P., Cardillo M., Rossini G. (2003). Incidence of cancer after kidney transplant: results from the North Italy transplant program. *Transplantation*.

[38] Penn I. (1993). Tumors after renal and cardiac transplantation. *Hematology/Oncology Clinics of North America*.

[39] Serraino D., Piselli P., Angeletti C. (2005). Risk of Kaposi's sarcoma and of other cancers in Italian renal transplant patients. *British Journal of Cancer*.

[40] Tatar E., Sarsik B., Gungor O. (2013). Multiple unrelated malignancies following renal transplantation: an evaluation of four cases. *Internal Medicine*.

[41] O'Donovan P., Perrett C. M., Zhang X. (2005). Azathioprine and UVA light generate mutagenic oxidative DNA damage. *Science*.

[42] Strocchi E., Iaffaioli R. V., Facchini G. (2004). Stop-flow technique for loco-regional delivery of high dose chemotherapy in the treatment of advanced pelvic cancers. *European Journal of Surgical Oncology*.

[43] Piscitelli P., Santoriello A., Buonaguro F. M. (2009). Incidence of breast cancer in Italy: mastectomies and quadrantectomies performed between 2000 and 2005. *Journal of Experimental and Clinical Cancer Research*.

[44] Insabato L., Siano M., Somma A., Gentile R., Santangelo M., Pettinato G. (2009). Extrapleural solitary fibrous tumor: a clinicopathologic study of 19 cases. *International Journal of Surgical Pathology*.

[45] Tortoriello A., Facchini G., Caponigro F. (1998). Phase I/II study of paclitaxel and vinorelbine in metastatic breast cancer. *Breast Cancer Research and Treatment*.

[46] Maurea S., Fusari M., Imbriaco M. (2012). Pitfalls in diagnostic imaging of cystic pancreatic masses: a case of true cystic lesion mimicking a mucinous cystadenoma. *Journal of the Pancreas*.

[47] Kauffman H. M., Cherikh W. S., McBride M. A., Cheng Y., Hanto D. W. (2007). Deceased donors with a past history of malignancy: an organ procurement and transplantation network/united network for organ sharing update. *Transplantation*.

[48] Santangelo M., Spiezia S., Clemente M., Renda A. (2009). Immunodeficiency and multiple primary malignancies. *Multiple Primary Malignancies*.

[49] Taioli E., Piselli P., Arbustini E. (2006). Incidence of second primary cancer in transplanted patients. *Transplantation*.

